# Serum Insulin-Like Growth Factor 1 Levels, Facture Risk Assessment Tool Scores and Bone Disorders in Patients with Primary Biliary Cholangitis

**DOI:** 10.3390/diagnostics12081957

**Published:** 2022-08-12

**Authors:** Chisato Saeki, Tsunekazu Oikawa, Kaoru Ueda, Masanori Nakano, Yuichi Torisu, Masayuki Saruta, Akihito Tsubota

**Affiliations:** 1 Division of Gastroenterology and Hepatology, Department of Internal Medicine, The Jikei University School of Medicine, 3-25-8 Nishi-shimbashi, Minato-ku, Tokyo 105-8461, Japan; 2 Division of Gastroenterology, Department of Internal Medicine, Fuji City General Hospital, 50 Takashima-cho, Fuji-shi, Shizuoka 417-8567, Japan; 3 Core Research Facilities, Research Center for Medical Science, The Jikei University School of Medicine, 3-25-8 Nishi-shimbashi, Minato-ku, Tokyo 105-8461, Japan

**Keywords:** primary biliary cholangitis, osteoporosis, fracture, fracture risk assessment tool

## Abstract

Insulin-like growth factor 1 (IGF-1) plays an important role in bone growth and maintenance, and its decreased levels are associated with bone disorders. This study aimed to evaluate the association of serum IGF-1 levels with osteoporosis, prevalent fractures and fracture risk based on the Fracture Risk Assessment Tool (FRAX) in patients with primary biliary cholangitis (PBC). This study included 127 consecutive patients with PBC. Based on the baseline serum IGF-1 levels, the participants were classified into the low (L)-, intermediate (I)- and high (H)-IGF-1 groups. According to the FRAX score, high fracture risk was defined as a 10-year major osteoporotic fracture probability (FRAX-MOF) ≥ 20% or a 10-year hip fracture probability (FRAX-HF) ≥ 3%. The serum IGF-1 levels were positively correlated with bone mineral density, and were negatively correlated with the FRAX-MOF/FRAX-HF. The L-IGF-1 group had the highest prevalence of osteoporosis (58.1%), prevalent fracture (48.4%) and high fracture risk (71.0%). Meanwhile, the H-IGF-1 group had the lowest prevalence of osteoporosis (9.7%), prevalent fracture (12.9%) and high fracture risk (9.7%). The prevalence of these events increased stepwise with decreasing serum IGF-1 levels. The cutoff values of IGF-1 for predicting osteoporosis, prevalent fracture and high fracture risk were 61.5 ng/mL (sensitivity/specificity, 0.545/0.894), 69.5 ng/mL (0.633/0.784) and 61.5 ng/mL (0.512/0.929), respectively. Serum IGF-1 levels were associated with bone disorders and the FRAX-derived fracture risk, and may be a useful indicator for initiating therapeutic intervention to prevent the incidence of fracture in patients with PBC.

## 1. Introduction

Primary biliary cholangitis (PBC) is an autoimmune liver disease characterized by the presence of highly specific antimitochondrial antibody, chronic nonsuppurative destructive cholangitis on histology and progressive cholestasis, leading to liver failure [1,2]. Osteoporosis and its associated fragility fractures are frequent extrahepatic complications of PBC [3,4,5,6]. A meta-analysis of eight studies (including 1643 patients with PBC and 10,291 controls) revealed that patients with PBC have a relative risk of 2.79 for osteoporosis [7]. Advanced age, low body mass index, advanced histological stage and sarcopenia are significant independent risk factors for osteoporosis [3,6,8]. Patients with PBC are at higher risk for any fractures than the general population, with a hazard ratio of 2.03 [9]. Specifically, patients with menopausal status, advanced age, short stature and osteoporosis are more likely to develop fractures [4]. Since bone disorders affect morbidity and quality of life, early diagnosis and treatment are crucial in preventing fracture [10]. However, appropriate assessment of osteoporosis and risk for fracture remains inadequate in real-world clinical settings.

The World Health Organization (WHO) advocated the Fracture Risk Assessment Tool (FRAX) in 2008 to assess the 10-year risk probabilities of major osteoporotic fracture (MOF) (e.g., proximal forearm, humerus and spinal) and hip fracture (HF) [11]. The FRAX yields the 10-year probabilities of MOF (FRAX-MOF) and HF (FRAX-HF) using a computer algorithm that comprises risk factors for osteoporotic fracture, including gender, age, previous fracture, bone mineral density (BMD) and diseases related to secondary osteoporosis (including chronic liver disease [CLD]). Therefore, the FRAX might be useful for assessing fracture risk in patients with PBC. The European Association for the Study of the Liver’s clinical guidelines for the management of PBC state that therapeutic intervention for osteoporosis should take into account the overall risk of fracture, which can be estimated by the FRAX [12]. The Bone Health and Osteoporosis Foundation states that pharmacologic treatment should be considered in patients with osteoporosis who have a T-score of ≤−2.5 and those with a reduced bone mass (T-score between −1.0 and −2.5) who have FRAX-MOF or FRAX-HF of ≥20% or ≥3%, respectively [13].

Insulin-like growth factor 1 (IGF-1), which is synthesized primarily in hepatocytes, regulates bone metabolism, and plays an important role in bone growth and maintenance [14,15]. Patients with CLD complicated by osteoporosis were reported to have lower serum IGF-1 levels than those without osteoporosis [6,16]. Serum IGF-1 levels were found to be negatively correlated with FRAX-MOF and FRAX-HF in patients with diabetes [17]. Low serum IGF-1 levels were associated with a high risk of incident hip and vertebral fractures in older men [18].

To date, no study has evaluated the FRAX-MOF and FRAX-HF in patients with PBC. This study aimed to assess the risk of fracture using the FRAX and the correlation between the FRAX and BMD in patients with PBC. Furthermore, the association of serum IGF-1 levels with bone disorders, the FRAX and high fracture risk was investigated.

## 2. Patients and Methods

### 2.1. Participants and Study Design

This cross-sectional study included 127 consecutive patients with PBC who visited the Jikei University School of Medicine (Tokyo, Japan) and Fuji City General Hospital (Shizuoka, Japan) between 2017 and 2021. The enrolled patients were 40–90 years of age as per the recommendation of the FRAX. All patients with osteoporosis were treatment-naïve at the time of entry. PBC was diagnosed according to the criteria proposed by the Japanese Intractable Hepatobiliary Disease Study Group [19]. A questionnaire and medical record data were used to investigate previous and family history of fracture, smoking status, glucocorticoid use, complications of rheumatoid arthritis (RA) and alcohol intake. The serum IGF-1 level was evaluated using an immunoradiometric assay (Fujirebio, Tokyo, Japan). The levels of serum zinc and Mac-2 binding protein glycosylation isomer (M2BPGi; liver fibrosis marker) were measured using routine laboratory methods. The current study was performed in accordance with the 2013 Declaration of Helsinki, and was approved by the ethics committee of the Jikei University School of Medicine (approval no. 28-194) and Fuji City General Hospital (approval no. 162). Written informed consent was obtained from all participants.

### 2.2. Evaluation of Osteoporosis and Vertebral Fracture

BMD at lumbar spine (L2–L4), femoral neck and total hip were assessed using dual-energy X-ray absorptiometry (PRODIGY; GE Healthcare Japan, Tokyo, Japan). Osteoporosis was diagnosed using the WHO criteria (T-score ≤ −2.5) [20]. Vertebral fractures were diagnosed by lateral radiography of the thoracolumbar spine using Genant’s semi-quantitative method [21].

### 2.3. Fracture Risk Assessment with the FRAX

FRAX scores (%) were calculated as FRAX-MOF and FRAX-HF using the Japan FRAX model with BMD (https://www.sheffield.ac.uk/FRAX/tool.aspx?country=3 accessed on 1 July 2022). The scoring model comprises the following risk factors for fragility fractures: gender, age, height, weight, previous history of fracture, parental history of HF, current smoking and drinking (≥3 units or 60 g/day), use of glucocorticoid (prednisolone at a dose of >5 mg/day for at least 3 months), presence of RA or diseases related to secondary osteoporosis (including CLD) and femoral neck BMD [11]. High fracture risk was defined as FRAX-MOF ≥ 20% or FRAX-HF ≥ 3% [13].

### 2.4. Grouping of Patients Based on Serum IGF-1 Levels

The median baseline IGF-1 level of all patients was 90 (interquartile range, 65–112) ng/mL. According to the first and third quartiles of IGF-1 levels, the patients were classified into three groups: the low (L)-IGF-1 group (<65 ng/mL); intermediate (I)-IGF-1 group (between 65 and 112 ng/mL); and high (H)-IGF-1 group (>112 ng/mL) (Figure S1).

### 2.5. Statistical Analysis

Categorical and continuous variables were presented as numbers with ratios and medians with interquartile ranges, respectively. The chi-squared test and the Mann–Whitney U test, as appropriate, were performed to assess between-group differences. The Jonckheere–Terpstra test and the Cochran–Armitage test were used to evaluate significant trends in continuous and categorical variables, respectively. The area under the receiver operating characteristic curve was constructed to estimate the optimal cutoff values of IGF-1 for predicting osteoporosis, prevalent fracture and high fracture risk. The SPSS Statistics version 27 (IBM Japan, Tokyo, Japan) was used for all statistical analyses. A *p*-value of <0.05 was considered statistically significant.

## 3. Results

### 3.1. Characteristics of the Patients

Table 1 shows the baseline characteristics of the 127 participants. The study cohort included 100 women (78.7%), with a median age of 66.0 (56.0–72.0) years. The prevalence of cirrhosis and osteoporosis was 9.4% (12/127) and 26.0% (33/127), respectively. Thirty (23.6%) patients had prevalent fractures at the following sites: spine, n = 22; rib, n = 5; distal radius, n = 2; proximal humerus, n = 1; hip, n = 1; pelvis, n = 1; and lower extremity, n = 3.

### 3.2. Clinical Characteristics of the High- and Non-High-Risk Groups

The median FRAX-MOF and FRAX-HF in overall patients were 7.7% (4.3–16.0%) and 1.3% (0.4–4.1%), respectively (Table 2). According to the FRAX criteria, 43 patients (33.9%) were at high risk for fractures (high-risk group), while the remaining 84 (66.1%) patients were not at high risk for fractures (non-high-risk group) (Table 1). In the high-risk group, the median FRAX-MOF and FRAX-HF were 19.0% (15.0–27.0%) and 6.3% (3.8–13.0%), respectively. Meanwhile, in the non-high-risk group, they were 5.1% (3.7–7.8%) and 0.7% (0.2–1.3%), respectively (Table 2). The high-risk group was older (*p* < 0.001) and had lower body height and weight (*p* < 0.001 for both) and higher prevalence of cirrhosis (*p* = 0.002) than the non-high-risk group. Regarding the laboratory data, the high-risk group had higher M2BPGi and lower IGF-1 levels than the non-high-risk group (*p* < 0.001 for both). The high-risk group had higher prevalence of osteoporosis (69.8% vs. 3.6%) and prevalent fracture (62.8% vs. 3.6%) than the non-high-risk group (*p* < 0.001 for both).

### 3.3. Prevalence of High Fracture Risk According to the Age Groups

**Figure 1:**
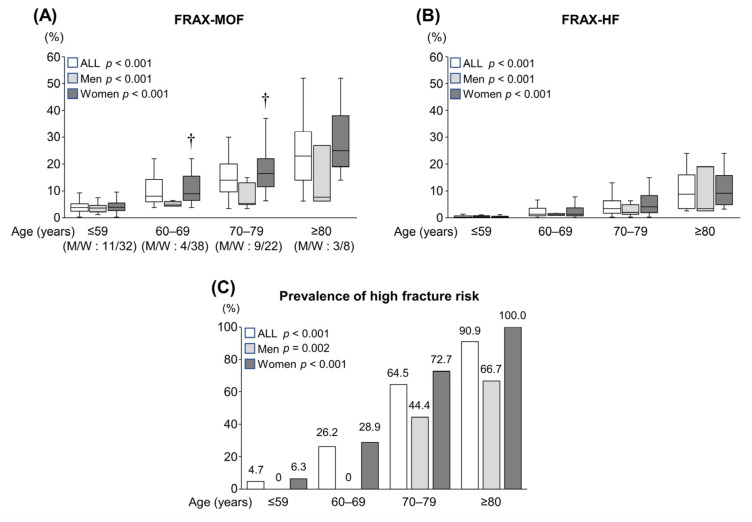
The 10-year probabilities of major osteoporotic fracture (FRAX-MOF) and hip fracture (FRAX-HF) and the prevalence of high fracture risk among the four age groups. (**A**) The FRAX-MOF significantly increased stepwise with advancing age. † Women had significantly higher FRAX-MOF than men. (**B**) The FRAX-HF significantly increased stepwise with advancing age. (**C**) The prevalence of high fracture risk significantly increased stepwise with advancing age. The numbers on each bar indicate the percentage of patients with high fracture risk. M/W, Men/Women; FRAX, Fracture Risk Assessment Tool. ^†^ *p* < 0.05.

The patients were stratified into four groups according to age (≤59, 60–69, 70–79 and ≥80 years). The FRAX-MOF and FRAX-HF in each group were estimated (Figure 1A,B). The median FRAX scores were 3.8% (2.4–5.2%) and 0.3% (0.1–0.7%) for the ≤59-year group, 8.1% (5.9–14.3%) and 1.4% (0.8–3.5%) for the 60–69-year group, 14.0% (9.7–20.0%) and 3.4% (1.7–6.3%) for the 70–79-year group and 23.0% (14.0–32.0%) and 8.8% (3.4–16.0%) for the ≥80-year group, respectively. Women had significantly higher FRAX-MOF than men in the 60–69- and 70–79-year groups. The FRAX-MOF and FRAX-HF in all patients, men and women, significantly increased stepwise with advancing age (Figure 1A,B; *p* < 0.001 for all). Accordingly, the prevalence of high fracture risk significantly increased stepwise with age (Figure 1C; *p* < 0.001 for all patients and women, *p* = 0.002 for men).

### 3.4. Correlations between BMD and FRAX Scores

**Figure 2:**
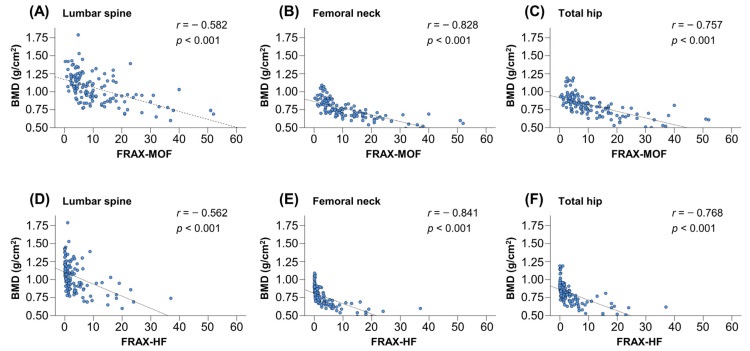
Correlations between bone mineral density (BMD) and the 10-year probabilities of major osteoporotic fracture (FRAX-MOF) and hip fracture (FRAX-HF). The FRAX-MOF was significantly correlated with BMD at lumbar spine (**A**), femoral neck (**B**) and total hip (**C**). The FRAX-HF was significantly correlated with BMD at lumbar spine (**D**), femoral neck (**E**) and total hip (**F**). FRAX, Fracture Risk Assessment Tool.

The FRAX-MOF and FRAX-HF were found to be significantly correlated with BMD at lumbar spine (*r* = −0.582 and −0.562, respectively), femoral neck (*r* = −0.828 and −0.841, respectively) and total hip (*r* = −0.757 and −0.768, respectively) (*p* < 0.001 for all; Figure 2A–F). 

### 3.5. Serum IGF-1 Levels According to Age Groups

We evaluated serum IGF-1 levels among the four age groups. The median serum IGF-1 levels were 107 (89–129) ng/mL for the ≤59-year group, 87 (68–107) ng/mL for the 60–69-year group, 67 (59–97) ng/mL for the 70–79-year group and 60 (42–91) ng/mL for the ≥80-year group. The serum IGF-1 levels significantly decreased stepwise with advancing age (Figure S2; *p* < 0.001).

### 3.6. Clinical Characteristics of the Three Groups Based on Serum IGF-1 Levels

**Figure 3:**
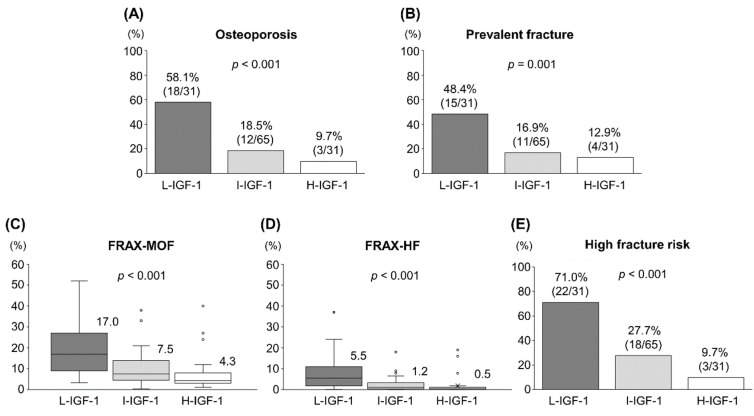
Clinical characteristics of the low-insulin-like growth factor 1 (L-IGF-1), intermediate-IGF-1 (I-IGF-1) and high-IGF-1 (H-IGF-1) groups. (**A**) The prevalence of osteoporosis significantly increased stepwise with decreasing serum IGF-1 levels. (**B**) The prevalence of prevalent fracture significantly increased stepwise with decreasing serum IGF-1 levels. (**C**) The 10-year probability of major osteoporotic fracture (FRAX-MOF) significantly increased stepwise with decreasing serum IGF-1 levels. The numbers on the upper right corner of each box indicate the median percentages in each group. (**D**) The 10-year probability of hip fracture (FRAX-HF) significantly increased stepwise with decreasing serum IGF-1 levels. The numbers on the upper right corner of each box indicate the median percentages in each group. (**E**) The prevalence of high fracture risk significantly increased stepwise with decreasing serum IGF-1 levels. FRAX, Fracture Risk Assessment Tool.

The proportions of L-IGF-1, I-IGF-1 and H-IGF-1 were 24.4% (31/127), 51.2% (65/127) and 24.4% (31/127), respectively (Table 3). The three groups significantly differed in terms of age (*p* < 0.001), body height (*p =* 0.033), disease duration (*p =* 0.020), prevalence of cirrhosis (*p* < 0.001), M2BPGi levels (*p* < 0.001) and BMD at lumbar spine, femoral neck and total hip (*p* < 0.001 for all). The L-IGF-1 group had the highest prevalence of osteoporosis (58.1%) and prevalent fracture (48.4%). Meanwhile, the H-IGF-1 group had the lowest prevalence of osteoporosis (9.7%) and prevalent fracture (12.9%) (Figure 3A,B). The prevalence of these bone disorders significantly increased stepwise with decreasing serum IGF-1 levels (*p* < 0.001 and *p* = 0.001, respectively). The median FRAX-MOF and FRAX-HF in the L-IGF-1 group were 17.0% (9.0–27.0%) and 5.5% (1.9–11.0%), respectively. Meanwhile, those in the H-IGF-1 group were 4.3% (3.1–8.0%) and 0.5% (0.1–1.2%), respectively (Figure 3C,D). The FRAX-MOF and FRAX-HF increased stepwise with decreasing serum IGF-1 levels (*p* < 0.001 for both). Accordingly, the prevalence of high fracture risk increased stepwise with decreasing serum IGF-1 levels (*p* < 0.001; Figure 3E).

### 3.7. Correlations between Serum IGF-1 Levels and FRAX Scores

**Figure 4:**
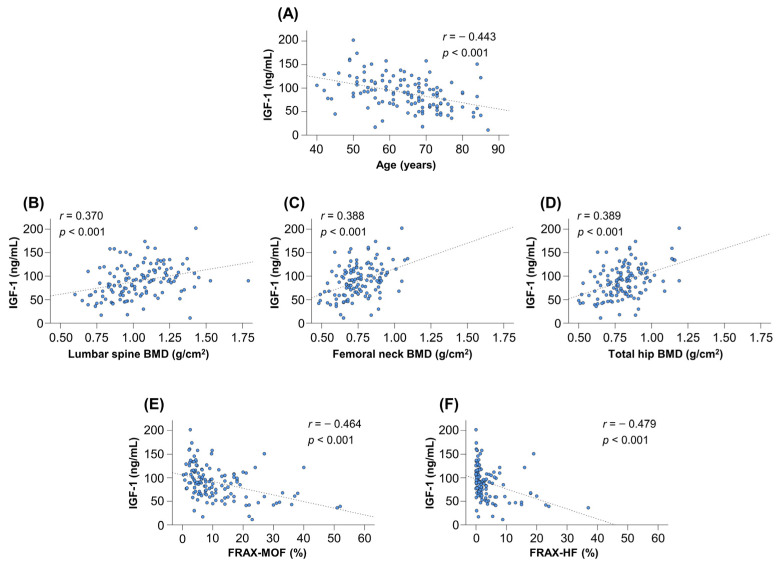
Correlations between serum insulin-like growth factor 1 (IGF-1) levels and clinical factors. The serum IGF-1 levels were significantly correlated with age (**A**), lumbar spine BMD (**B**), femoral neck BMD (**C**) and total hip BMD (**D**). The serum IGF-1 levels were significantly correlated with the 10-year probabilities of major osteoporotic fracture (FRAX-MOF) (**E**) and hip fracture (FRAX-HF) (**F**). BMD, bone mineral density; and FRAX, Fracture Risk Assessment Tool.

The serum IGF-1 levels were significantly correlated with age (*r* = −0.443), BMD at lumbar spine (*r* = 0.370), femoral neck (*r* = 0.388) and total hip (*r* = 0.389) (*p* < 0.001 for all; Figure 4A–D). There were significant correlations between serum IGF-1 levels and the FRAX-MOF/FRAX-HF (*r* = −0.464 and −0.479, respectively) (*p* < 0.001 for both; Figure 4E,F).

### 3.8. Optimal Cutoff Values of IGF-1 for Predicting Osteoporosis, Prevalent Fracture and High Fracture Risk

**Figure 5:**
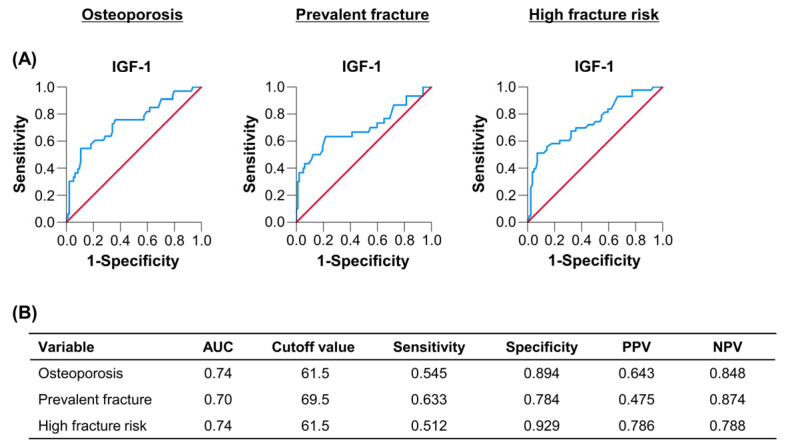
(**A**) The receiver operating characteristic curve analysis of serum insulin-like growth factor 1 (IGF-1) for predicting osteoporosis, prevalent fracture and high fracture risk. (**B**) The cutoff values of IGF-1 for predicting these events were 61.5 ng/mL for osteoporosis, 69.5 ng/mL for prevalent fracture and 61.5 ng/mL for high fracture risk. AUC, area under the curve; NPV, negative predictive value; and PPV, positive predictive value.

Figure 5 shows the cutoff values and performances of IGF-1 for predicting osteoporosis, prevalent fracture and high fracture risk. The cutoff values of IGF-1 for predicting these bone disorders were 61.5 ng/mL (area under the curve: 0.74; sensitivity/specificity: 0.545/0.894), 69.5 ng/mL (0.70; 0.633/0.784) and 61.5 ng/mL (0.74; 0.512/0.929), respectively (Figure 5A,B).

### 3.9. Fracture Risk between the Cirrhosis and Non-Cirrhosis Groups

The cirrhosis group had significantly higher prevalence of osteoporosis (22.6% vs. 58.3%; *p* = 0.007) and prevalent fracture (20.0% vs. 58.3%; *p* = 0.003) than the non-cirrhosis group (Figure S3A,B). Conversely, the cirrhosis group had significantly lower serum IGF-1 levels than the non-cirrhosis group (*p* < 0.001; Figure S3C). The cirrhosis group had significantly higher FRAX-MOF (*p* = 0.004), FRAX-HF (*p* < 0.001) and prevalence of high fracture risk (29.6% vs 75.0%; *p* = 0.002) than the non-cirrhosis group (Figure S3D–F).

## 4. Discussion

Bone disorders, including osteoporosis and its associated bone fractures, are the common extrahepatic complications in patients with PBC, and are often referred to as hepatic osteodystrophy [22]. Bone disorders can lead to impaired physical function and poor prognosis [23]. Therefore, it is important to evaluate bone mass and fracture risk and initiate osteoporosis treatment as early as possible to prevent fracture. Recently, several academic societies have addressed or discussed when to initiate osteoporosis treatment using the FRAX [12,13]. Meanwhile, IGF-1 plays a vital role in bone growth and homeostasis, and its decreased levels are associated with bone disorders [6,14,15,16,17,18]. Hence, serum IGF-1 levels may be useful for predicting fracture risk. This is the first study to estimate the FRAX-MOF and FRAX-HF and evaluate the association of serum IGF-1 levels with the FRAX scores and high fracture risk in patients with PBC.

The FRAX-MOF and FRAX-HF in patients with PBC were 7.7% and 1.3%, respectively, in this study, and the prevalence of high fracture risk was 33.9%. Notably, the FRAX-MOF and FRAX-HF significantly increased with decreasing serum IGF-1 levels and were significantly and negatively correlated with serum IGF-1 levels. Accordingly, the prevalence of high fracture risk significantly increased with decreasing serum IGF-1 levels. The cutoff values of serum IGF-1 for predicting osteoporosis, prevalent fracture and high fracture risk were 61.5, 69.5 and 61.5 ng/mL, respectively. A study on patients with diabetes reported that serum IGF-1 levels were positively correlated with BMD at femoral neck and total hip and were negatively correlated with the FRAX-MOF and FRAX-HF [17]. Another study on patients with diabetes reported that serum IGF-1 levels and BMD at lumbar spine and femoral neck were independent factors associated with prevalent vertebral fracture [24]. The cutoff values for predicting prevalent vertebral fracture were 127 ng/mL and −1.67/−1.24 in men and 127 ng/mL and −1.78/−2.02 in postmenopausal women, respectively. In the other study of older men, low serum IGF-1 levels were associated with high risk of incident vertebral and hip fractures [18]. The risk of these fractures increased by 40% and 45%, respectively, per standard deviation decrease in serum IGF-1. Serum IGF-1 levels of <112 ng/mL (median) were inversely correlated with the annual fracture incidence. In addition, the group with serum IGF-1 levels of ≤85 ng/mL (25th percentile) had the highest risk of fractures. These results indicated that serum IGF-1 levels could be useful for predicting bone disorders and high fracture risk. However, the cutoff values vary according to underlying diseases or conditions, and are likely to be lower in patients with CLD than in those without liver dysfunction.

Circulating IGF-1 is produced mainly in hepatocytes, and its level decreases with progression of liver disease [25]. This study revealed that patients with cirrhosis had significantly lower serum IGF-1 levels than those without cirrhosis. In addition, patients with cirrhosis had higher FRAX-MOF and FRAX-HF and higher prevalence of osteoporosis, prevalent fracture and high fracture risk than those without cirrhosis. In one study on PBC, patients with histologic stage 3 or 4 had a 5.4-fold-increased risk of osteoporosis than those with stage 1 or 2 [8]. In another study of men aged ≥50 years, nonalcoholic fatty liver disease with fibrosis (but not without fibrosis) was significantly associated with increased FRAX-MOF and FRAX-HF [26]. From another perspective, IGF-1 was reported to induce senescence of hepatic stellate cells and inhibited the progression of hepatic fibrosis in nonalcoholic steatohepatitis and cirrhotic mouse models [27]. A study on children with end-stage liver disease showed that low BMD was associated with low serum IGF-1 levels and liver transplantation led to significant improvement in serum IGF-1 levels and BMD [28]. Given that IGF-1 signaling promotes osteoblast and osteoclast cell differentiation, increases type I collagen transcription and inhibits collagenase synthesis, IGF-1 plays an essential role in bone remodeling and maintenance [14]. Accordingly, decreased IGF-1 levels could lead to low bone mass, bone disorders and high fracture risk, as suggested in this study. In addition, IGF-1 is also involved in muscle protein synthesis and activation of satellite cells through mammalian target of the rapamycin signaling [29]. We have previously reported that low IGF-1 levels were associated with sarcopenia (defined as a loss of skeletal muscle mass and strength) and impaired physical performance in patients with cirrhosis [30]. These conditions may increase the risk of falls and fractures. Taken together, IGF-1 plays a crucial role in the musculoskeletal system and decreased IGF-1 levels could result in musculoskeletal disorders and high fracture risk. Therefore, we should be cautious about the risk of fractures particularly in patients with advanced-stage liver disease who have low serum IGF-1 levels.

This study has some limitations. First, this cohort did not include healthy controls or individuals without liver dysfunction. Second, since PBC is a relatively rare disease, the sample size was not large enough to evaluate the impact of serum IGF-1 levels on the risk of fracture in the subgroup analysis. Finally, this was a cross-sectional study; thus, the association between FRAX scores and fractures was not prospectively investigated. A large-scale, prospective study should be introduced to validate the usefulness of FRAX and serum IGF-1 levels for predicting fractures in patients with PBC and age-matched controls without liver dysfunction.

## 5. Conclusions

This study demonstrated that approximately one-third of patients with PBC were at high risk for fracture. The prevalence of bone disorders (osteoporosis and prevalent fracture) and the 10-year FRAX-derived probability of MOF and HF (including high fracture risk) increased with decreasing serum IGF-1 levels. Conversely, BMD values increased with increasing serum IGF-1 levels. The pros and cons and timing of therapeutic intervention should be established by certain indicators, such as the FRAX and serum IGF-1 level, to rationally prevent the development of osteoporosis and fracture in patients with PBC.

## Figures and Tables

**Table 1:** Comparison of clinical characteristics between patients with and without high fracture risk based on the FRAX.

Variable	All Patients	FRAX	FRAX	*p* Value
		High Risk	Non-High Risk	
Patients, n (%)	127	43 (33.9)	84 (66.1)	0.150
Women, n (%)	100 (78.7)	37 (86.0)	63 (75.0)	<0.001
Age (years)	66.0 (56.0–72.0)	73.0 (69.0–77.0)	60.0 (53.3–68.0)	<0.001
Body height (cm)	157.0 (150.0–162.0)	151.3 (145.0–156.0)	158.5 (153.3–164.9)	<0.001
Body weight (kg)	54.0 (48.6–63.2)	49.5 (44.1–55.4)	56.6 (51.3–65.2)	0.418
BMI (kg/m^2^)	22.2 (20.3–24.7)	22.2 (20.1–24.7)	22.3 (20.4–24.7)	0.048
Disease duration (years)	2.0 (1.0–8.0)	4.0 (1.0–9.0)	2.0 (1.0–6.0)	0.002
Cirrhosis, n (%)	12 (9.4)	9 (20.9)	3 (3.6)	0.514
Smoking, n (%)	31 (24.4)	9 (20.9)	22 (26.2)	0.486
Alcohol intake, n (%)	9 (7.1)	4 (9.3)	5 (6.0)	<0.001
M2BPGi (C.O.I)	0.85 (0.62–1.28)	1.24 (0.68–1.79)	0.73 (0.57–0.99)	<0.001
IGF-1 (ng/mL)	90 (65–112)	61 (46–97)	96 (77–118)	<0.001
Lumbar spine BMD (g/cm^2^)	1.03 (0.90–1.19)	0.90 (0.78–0.96)	1.09 (0.98–1.22)	<0.001
Lumbar spine T score	−1.00 (−1.96–−0.10)	−1.92 (−2.90–−1.20)	−0.58 (−1.40–0.29)	<0.001
Femoral neck BMD (g/cm^2^)	0.74 (0.67–0.86)	0.65 (0.59–0.69)	0.82 (0.74–0.90)	<0.001
Femoral neck T score	−1.76 (−2.34–−1.00)	−2.60 (−3.11–−2.20)	−1.23 (−1.80–−0.52)	<0.001
Total hip BMD (g/cm^2^)	0.80 (0.72–0.89)	0.69 (0.62–0.77)	0.85 (0.79–0.93)	<0.001
Total hip T score	−1.31 (−1.90–−0.80)	−2.20 (−2.69–−1.50)	−0.98 (−1.48–−0.32)	<0.001
Osteoporosis, n (%)	33 (26.0)	30 (69.8)	3 (3.6)	<0.001
Prevalent fracture, n (%)	30 (23.6)	27 (62.8)	3 (3.6)	

Values are shown as median (interquartile range) or number (percentage). Statistical analysis was performed using the chi-squared test or the Mann-Whitney U test, as appropriate. FRAX, Fracture Risk Assessment Tool; BMD, bone mineral density; BMI, body mass index; IGF-1, insulin-like growth factor 1; and M2BPGi, Mac-2 binding protein glycosylation isomer.

**Table 2:** The 10-year probabilities of major osteoporotic and hip fractures based on the FRAX.

10-Year Probability of Fracture	All Patients	FRAX	FRAX
		High Risk	Non-High Risk
Major osteoporotic fracture (%)	7.7 (4.3–16.0)	19.0 (15.0–27.0)	5.1 (3.7–7.8)
Hip fracture (%)	1.3 (0.4–4.1)	6.3 (3.8–13.0)	0.7 (0.2–1.3)

FRAX, Fracture Risk Assessment Tool.

**Table 3:** Characteristics of the three groups classified based on the baseline IGF-1 levels.

Variable	L-IGF-1	I-IGF-1	H-IGF-1	*p* Value
Patients, n (%)	31 (24.4)	65 (51.2)	31 (24.4)	
Women, n (%)	26 (83.9)	53 (81.5)	21 (67.7)	0.220
Age (years)	73.0 (68.0–77.0)	65.0 (56.0–71.5)	58.0 (51.0–64.0)	<0.001
Body height (cm)	152.5 (144.2–160.7)	152.0 (157.0–160.1)	159.8 (151.0–169.0)	0.033
Body weight (kg)	52.6 (45.0–57.0)	54.9 (48.3–62.7)	56.0 (49.8–68.0)	0.147
BMI (kg/m^2^)	22.2 (20.4–24.0)	22.3 (19.5–24.6)	21.5 (20.3–25.7)	0.993
Disease duration (years)	7.0 (1.0–13.0)	2.0 (1.0–7.0)	2.0 (1.0–4.0)	0.020
Cirrhosis, n (%)	9 (29.0)	3 (4.6)	0 (0.0)	<0.001
Smoking, n (%)	6 (19.4)	17 (26.2)	8 (25.8)	0.752
Alcohol intake, n (%)	1 (3.2)	4 (6.2)	4 (12.9)	0.304
M2BPGi (C.O.I)	1.33 (0.89–2.39)	0.74 (0.59–1.11)	0.74 (0.55–0.99)	<0.001
IGF-1 (ng/mL)	48 (42–59)	90 (77–100)	129 (118–151)	<0.001
Lumbar spine BMD (g/cm^2^)	0.91 (0.78–1.05)	1.07 (0.94–1.22)	1.12 (0.95–1.21)	<0.001
Lumbar spine T score	−1.80 (−2.90–−1.00)	−0.80 (−1.78–0.26)	−0.38 (−1.20–0.26)	<0.001
Femoral neck BMD (g/cm^2^)	0.66 (0.60–0.75)	0.75 (0.69–0.88)	0.82 (0.72–0.91)	<0.001
Femoral neck T score	−2.50 (−2.99–−1.66)	−1.70 (−2.20–−0.90)	−1.26 (−2.06–−0.40)	<0.001
Total hip BMD (g/cm^2^)	0.72 (0.63–0.83)	0.80 (0.75–0.89)	0.85 (0.80–0.94)	<0.001
Total hip T score	−1.90 (−2.60–−1.20)	−1.31 (−1.75–−0.76)	−0.90 (−1.50–−0.30)	<0.001
Osteoporosis, n (%)	18 (58.1)	12 (18.5)	3 (9.7)	<0.001
Prevalent fracture, n (%)	15 (48.4)	11 (16.9)	4 (12.9)	<0.001
10-year probability of MOF, (%)	17.0 (9.0–27.0)	7.5 (4.5–14.0)	4.3 (3.1–8.0)	<0.001
10-year probability of HF, (%)	5.5 (1.9–11.0)	1.2 (0.4–3.3)	0.5 (0.1–1.2)	<0.001
High fracture risk, n (%)	22 (71.0)	18 (27.7)	3 (9.7)	<0.001

Values are shown as median (interquartile range) or number (percentage). Statistical analysis was performed using the chi-squared test or the Kruskal-Wallis test, as appropriate. BMD, bone mineral density; BMI, body mass index; HF, hip fracture; IGF-1, insulin-like growth factor 1; M2BPGi, Mac-2 binding protein glycosylation isomer; and MOF, major osteoporotic fracture.

## Data Availability

The data presented in this study are available on request from the corresponding author. The data are not publicly available due to patients’ privacy.

## Supplementary Materials

The following supporting information can be downloaded at https://www.mdpi.com/article/10.3390/diagnostics12081957/s1: Figure S1, Classification according to baseline serum insulin-like growth factor 1 (IGF-1) levels; Figure S2, Comparison of serum insulin-like growth factor 1 (IGF-1) levels among the four age groups; and Figure S3, Clinical characteristics of patients with cirrhosis and non-cirrhosis.
