# Supplementary material for: Serum Insulin-Like Growth Factor 1 Levels, Facture Risk Assessment Tool Scores and Bone Disorders in Patients with Primary Biliary Cholangitis
Source: Diagnostics (Basel). 2022 Aug 12;12(8):1957. doi: 10.3390/diagnostics12081957 (PMC9407172; doi:10.3390/diagnostics12081957)
Supplement: Supplementary file 1 [file diagnostics-12-01957-s001.zip › diagnostics-1833223-supplementary.pdf]

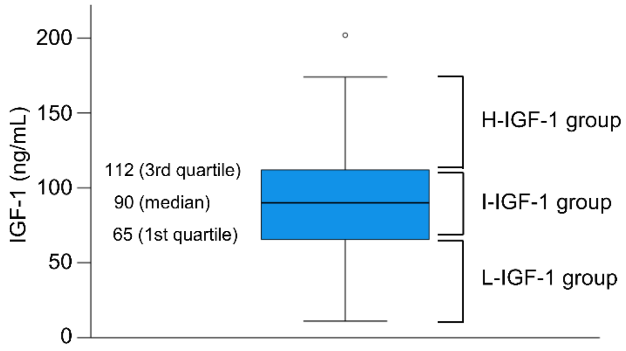

Figure S1. Classification according to baseline serum insulin-like growth factor 1 (IGF-1) levels. The median (interquartile range) serum IGF-1 level was 90 (65–112) ng/mL. According to the IGF-1 levels in the first and third quartiles, the 127 patients were classified into the three groups: the low-IGF-1 (L-IGF-1) group (<65 ng/mL), intermediate-IGF-1 (I-IGF-1) group (between 65 and 112 ng/mL), and high-IGF-1 (H-IGF-1) group (>112 ng/mL).

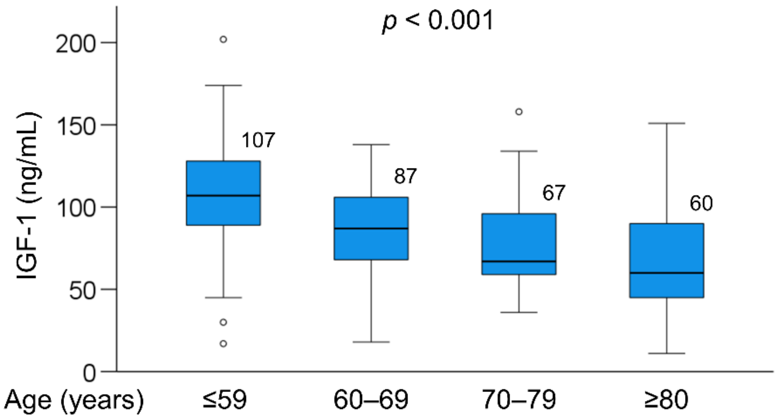

Figure S2. Comparison of serum insulin-like growth factor 1 (IGF-1) levels among the four age groups. The serum IGF-1 levels significantly decreased stepwise with advancing age. The numbers on the upper right corner of each box indicate the median values in each group.

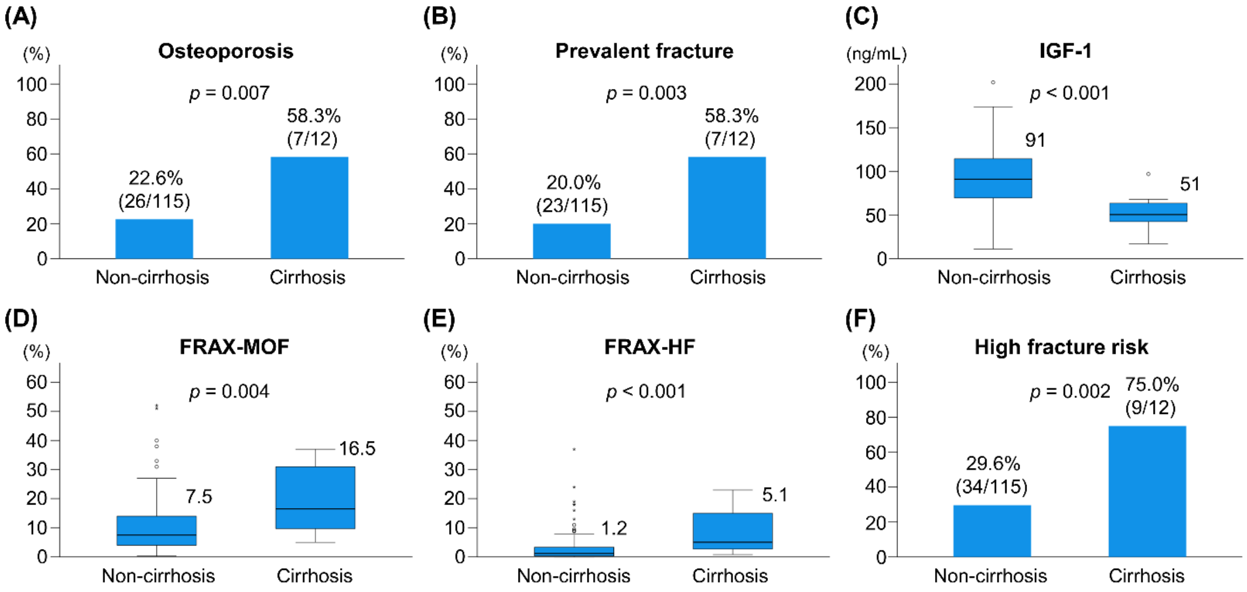

Figure S3. Clinical characteristics of patients with cirrhosis and non-cirrhosis. (**A**,**B**) The cirrhosis group had significantly higher prevalence of osteoporosis and prevalent fracture than the non-cirrhosis group. (**C**) The cirrhosis group had significantly lower serum insulin-like growth factor 1 (IGF-1) levels than the non-cirrhosis group. The numbers on the upper right corner of each box indicate the median values in each group. (**D**,**E**) The cirrhosis group had significantly higher 10-year probabilities of major osteoporotic fracture (FRAX-MOF) and hip fracture (FRAX-HF) than the non-cirrhosis group. The numbers on the upper right corner of each box indicate the median percentages in each group. (**F**) The cirrhosis group had significantly higher prevalence of high fracture risk than the non-cirrhosis group. FRAX, Fracture Risk Assessment Tool.
